# The Variation of Heavy Metals Bioavailability in Sediments of Liujiang River Basin, SW China Associated to Their Speciations and Environmental Fluctuations, a Field Study in Typical Karstic River

**DOI:** 10.3390/ijerph18083986

**Published:** 2021-04-10

**Authors:** Yupei Hao, Xiongyi Miao, Hongwei Liu, Dan Miao

**Affiliations:** 1 Key Laboratory of Karst Dynamics, MNR&GZAR, Institute of Karst Geology, CAGS, Guilin 541004, China; yphao66@126.com; 2 The Second Engineering Investigation Institute of Guizhou Bureau of Geology and Mineral Exploration and Development, Zunyi 563000, China; 3 Anhui Province Key Laboratory of Polar Environment and Global Change, Department of Environmental Science and Engineering, University of Science and Technology of China, Hefei 230026, China; lhw2016@mail.ustc.edu.cn; 4 Department of Chemistry and Environmental Engineering, Wuhan Bioengineering Institute, Wuhan 430415, China; miaodan23@126.com

**Keywords:** heavy metals, sediments, bioavailability, speciations, Liujiang River Basin

## Abstract

The bioavailability of heavy metals (HMs) in sediments is closely related to the security of the aquatic environment, but their impacts are poorly researched, particularly in karstic rivers. Therefore, Liujiang River Basin was taken as an example in this study. Seven HMs were analyzed to determine the bioavailability and speciations of HMs in sediments. Moreover, the impacts of environmental factors on HMs were identified. The obtained results suggested that HMs in the sediments are all within their permissible exposure limit (PEL), but Cd and Zn are significantly higher than the soil baseline. Most HMs were found to be in a residual fraction, while their exchangeable fraction was found to be in an extremely low ratio. HMs in bioavailable parts are significantly higher than in the exchangeable and carbonate-bound phases but lower than in the non-residual phase, which demonstrated that HM bioavailability is not confined to the exchangeable and carbonate-bound phases. The correlation coefficients commonly decreased with decreasing speciation ratios, which suggested that the overall bioavailability of metals should be determined by speciation ratios instead of speciations themselves. Noteworthily, most HMs in the residual form were found to be significantly correlated with their overall bioavailability, which highlighted the potential bioavailability of residual form. The non-correlations between pH, electrical conductivity (EC), total dissolved solids (TDS), and HM bioavailability suggested that HMs in the carbonate-bound phase are stable and unsusceptible to environmental variations, while the significant correlations between redox potential (Eh), turbidity, organic matter (OM), main grain size (Mz), and HM bioavailability suggested that HMs in the reducible and oxidizable forms are susceptible to environmental fluctuations. Therefore, the variation of HM bioavailability in karstic rivers is largely regulated by their reducible and oxidizable forms instead of their carbonate-bound form.

## 1. Introduction

Rivers are critical channels for the regulation and storage of water resources. However, with the development of industry and agriculture, they have gradually turned into vital channels for sewage emission. Based on previous studies, rivers are commonly found to be contaminated with single or several heavy metals (HMs) in various degrees across the world [1,2,3,4,5]. Due to the low dissolubility of HMs [6], these released exogenous HMs eventually settle down and preserve in sediments, which prolongs their toxicological effect on aquatic biotas [7]. Given that the ecological effect of HMs is closely related to their bioavailable parts in sediments [8,9], not their total content, it is essential to pay more attention to the bioavailability of HMs in sediments.

HMs can preserve as a variety of chemical forms in sediments, which is closely related to the active substances they combine with, e.g., organic matter, sulfide, carbonate, and Fe/Mn oxy-hydroxide [10,11,12]. Multiple analysis methods have been proposed to simplify the existing forms of HMs [13,14,15,16], the most widely used of which was proposed by Tessier and included exchangeable, carbonate-bound, reducible, oxidizable, and residual fractions [14]. Given the higher bioavailability of HMs in exchangeable fractions and carbonate-bound fractions [17,18], previous studies mainly aimed to degrade the threat of HM contamination via triggering HMs in these weakly bonded forms transform into their reducible and oxidizable fractions [19,20,21], which were commonly considered to be strongly bonded [17,18]. Though HMs in reducible and oxidizable fractions are less bioavailable [22], more and more studies have found that these HM forms can still affect HM bioaccumulation among aquatic biotas [23,24,25,26]—even the relatively inert residual form of HMs can release metallic/metalloid ions under the actions of many bacteria [24,25,27,28]. All of this suggested that these less bioavailable forms of HMs have potential bioavailability, so impacts on the overall bioavailability of HMs in sediments should be not overlooked.

In karst areas, the weathering of carbonate rocks is widespread, such that carbonate ions that are sourced from carbonate rock weathering are constantly and largely injected into rivers with the converging of surface runoffs, which results in the excessive existence of carbonate ions in rivers [17,29,30,31,32]. With the existence of affinities between carbonate ions and metallic/metalloid ions [33,34], exogenous HMs are confirmed to be able to largely preserve in the carbonate-bound form [17]; however, the carbonate-bound form is a weakly bonded form of HMs [33,34] that is less stable and susceptible to environmental fluctuations [17]. The environmental fluctuations in karstic rivers were confirmed to be drastic [35], particularly in monsoons, so the overall bioavailability of HMs in sediments is more likely regulated by carbonate-bound HMs. However, with the influence of other HM speciations and various environmental fluctuations, it is hard to determine the regulation level of carbonate-bound HMs on the overall bioavailability of HMs in rivers. Therefore, it is essential to clarify the regulation of HM speciations on the overall bioavailability of HMs (especially under environmental fluctuations), which could promote the identification of ecological risk in rivers.

The watershed of Liujiang River is a typical subtropical karst catchment in southwest China. It is located in Liuzhou city, which is a regional economic center and the largest industrial city in Guangxi Province. Extensive industrial operations have made Liuzhou a key city with a huge discharge of effluent. The annual emission of wastewater is higher than 350 million tons, more than 80% of which come from industrial operations related to the metal smelting, chemical, foods, and paper industries [36,37]. The wastewater discharging into the watershed of Liujiang River eventually converges into Liujiang River and its tributary of Luoqingjiang River, which makes metals become the main pollutants in Liujiang River and Luoqingjiang River [36,37,38]. In recent years, strict restrictions on sewage discharging have been enforced in these waterways to restore their ecology and secure water quality, such that a great improvement of water quality was confirmed in a previous study that found that the HM content in water is significantly superior to the primary standard of surface water quality [39]. However, the ubiquitous contamination of Cr, Cd, and Zn among fish disclosed in recent studies highlights the potential HM bioaccumulation from sediments [36,37,38]. The drastic precipitation in monsoons may aggravate the fluctuation of water chemistry in the Liujiang River Basin and then significantly elevate the bioconversion of HMs in sediments, which is definitely not conducive to the safety of an aquatic environment. Therefore, it is essential to ascertain the bioavailability of HMs in sediments and their impacts in order to take a deep insight into the long-term effect of HM contamination in local rivers. For this reason, this study intended to (1) investigate the bioavailability and speciations of heavy metals in sediments, (2) determine the impacts of HM speciations in sediment on their bioavailability, and (3) to gauge the impacts of environmental fluctuation on HM bioavailability. To the best of our knowledge, this is the very first study to attempt to establish the linkage of the speciations of HMs in sediment to their bioavailability, which will not only improve the identification of ecological and environmental risks but also exert a positive impact on environmental monitoring, control, and governance in rivers.

## 2. Materials and Methods

### 2.1. The Description of the Study Area and Field Sampling

Liujiang River Basin is located in Liuzhou City, China, with an area of 58,398 ${\mathrm{km}}^{2}$, the most vital surface runoffs of which are considered to be Liujiang River and Luoqingjiang River. Among them, Liujiang River is the largest river in Liuzhou City with a total length of 272 km; it starts with the confluence of Longjiang and Duliujiang Rivers in Fengshan Town and then flows through the most functional areas of Liuzhou City, including main industrial areas, residential areas, and the city center. Though the watershed of Luoqingjiang River mainly includes rural areas without significant industrial operation, though some industrial parks and industrial areas in the east suburban areas of Liuzhou City are also recognized in its watershed [40]. The wide distribution of carbonate and karst landforms breed a intact karstic groundwater system in Liuzhou River Basin so that the continuous recharge of alkaline groundwater from the karstic groundwater system to Liujiang River and Luoqingjiang River comes out, which also makes the water alkaline in these rivers all year round.

**Figure 1:**
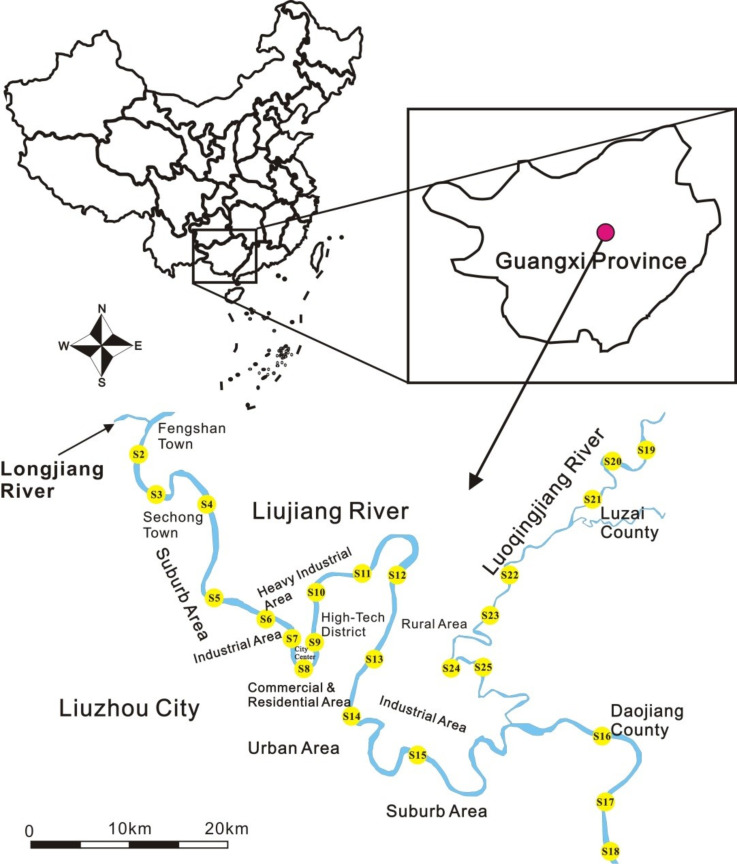
Sample collection distribution of Liujiang River Basin.

The sample collection was conducted from 12 to 18 May 2019—one week after two-week continuous rainfall to avoid the possible weakening of HM conversion. Twenty-four sampling sites were selected along the Liujiang River and its tributary Luoqingjiang River (Figure 1). The sediment samples were collected by using a grab sampler. Three subsamplers were collected within 100 m and combined into one with the weight of 1 kg in each sampling site. A total of 24 test samples of surface sediment were obtained from S2 to S25. After collection, 5 mL of nitric acid were added in water samples, which were then stored in 500 mL polypropylene plastic bottles while the sediment samples were preserved in polythene self-sealing bags. All of these samples were transported at −20 °C until further processing. The physicochemical parameters of the aquatic environment, including pH, redox potential (Eh), dissolved oxygen (DO), electrical conductivity (EC), total dissolved solids (TDS), and turbidity, were simultaneously determined with a multi-parameter water quality probe with the aims of exploring the behaviors of heavy metals in bioavailable parts during environmental fluctuations.

### 2.2. Sample Preparation and Analysis

The sediment samples were prepared in accordance with a previous study [6,41]. They were freeze-dried (−80 °C) 72 h to a constant weight and sieved through a 0.15 mm nylon mesh; then, 0.2 g of the sediment samples were directly digested in a solution of HNO_3_, HCl, and HF (5:4:1 *v/v*, 140 °C, and 6 h) for the total HM content testing.

The distribution of HMs in different geochemical fractions was obtained by using the sequential extraction procedure (SEP)—described in detail in [23,34,42]. On the basis of the SEP, HMs could be divided into exchangeable (Fr1), carbonate-bound (Fr2), reducible (Fr3), oxidizable (Fr4), and residual fractions (Fr5). To obtain the percent recovery of the HMs using the adopted SEP, the total HM concentrations were compared with the total concentrations of the HMs in the five SEP-derived fractions.

The extraction of bioavailable HMs was used to determine the bioavailability of HMs in this study. Despite many extraction agents being proposed to gauge the bioavailable parts of HMs in previous studies [8,43], HNO_3_ with a concentration of 0.75–1.0 mol∙L^−1^ was considered to be the most effective way to extract the bioavailable parts of HMs in sediments [8]. The detailed procedure was that a 50 mg sediment sample was digested by 50 mL of HNO_3_ (0.75–1.0 mol∙L^−1^) and hermetically shaken for 16 h. The supernatant was taken and filtered with 0.45 μm syringe-driven filters after centrifuging for further testing.

Cd, Cr, Cu, Pb, and Zn were analyzed by inductively coupled plasma mass spectrometry (ICP-MS, Thermo X series), while Hg and As were measured with the atomic fluorescence spectrometry (AFS-920). Organic matter was tested by an elemental analyzer (Vario EL-III), and sediment particle size was tested by a laser particle size analyzer (Mastersizer 2000).

### 2.3. Quality Assurance and Quality Control

Standard and blank samples were randomly inserted in the test process of each batch of samples. All measurements were minus the average of blank samples as the final sample test values, and a parallel sample was set for each sample, with the average value as the final result. The standard materials purchased from the Chinese Academy of Sciences were used in the sequential extraction process to ensure the accuracy of the extraction results, and the sample recovery ratio was calculated after the test. The recovery rates of total HM contents were reported to be between 95% and 105%, while the average recovery percentage of the total concentrations in the five fractions of HMs compared with the total HM concentrations was 93.68% ± 5.52%, which qualified Quality Assurance/Quality Control (QA/QC) compliance of DZ/T 0130-2006.

### 2.4. Statistical Analysis

The corresponding data of various environmental parameters, metal speciation, and bioavailability in each sampling site were incorporated into correlation analysis to explore their interactions, which was conducted with the help of SPSS 20. *p* < 0.05 indicated a significant difference in the data. The histograms and scatter diagrams were originally output with Origin Pro 8, but the further processing of these figures was done totally in Coreldraw X4.

## 3. Results and Discussion

### 3.1. The Properties in Sediments and Their Overlying Water

The properties of overlying water and sediment are given in Table 1 and Figures S1 and S2. The pH was found to range from 6.79 to 8.48 (with an average of 7.81) and was generally weakly alkaline, which was consistent with the characteristics of rivers in karst regions [44]. DO ranged from 6.22 to 8.43 mg/L, showing the water to be oxygen-rich. In contrast, the Eh, EC, TDS, and turbidity presented larger spatial differences. The variation of Eh was 94.89~161.10 mV, expressing an overall trend of decline from upstream to downstream. EC and TDS reflected the contents of soluble ions and soluble substances in water, respectively. The same spatial variation between them was found in this study. Turbidity is an index of suspended particulate matter in water (e.g., mineral particles, inorganic and organic colloids, polymer, bacteria, and algae), the range of which was observed to be within 8.13~28.10 NTU. Sediments were mainly comprised of fine particles (clay and silt, <63 μm) and dominated by silt (4~63 μm), with the mean proportion of 74.05%, while the average proportions of clay (<4 μm) and sand (63~125 μm) were 22.16% and 3.79%, respectively. The average value of organic matter (OM) in surface sediments was 0.72% (Figure 1), which was relatively low.

### 3.2. The Concentration and Their Speciations of Heavy Metals in Sediments

**Figure 2:**
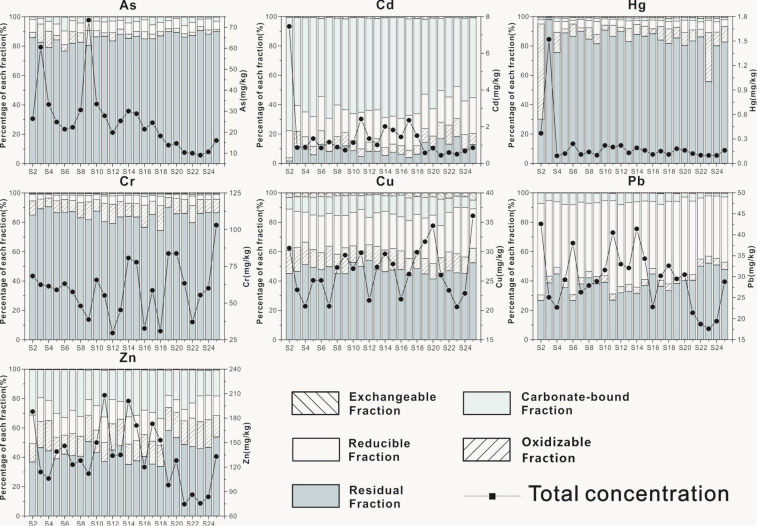
Heavy metal levels (mg/kg dry weight) in sediments with different fractions.

The concentrations and speciations of heavy metals in sediments are shown in Table 2, Figure 2, and Table S1, respectively. The concentrations of HMs were in the decreasing order as follows: Zn > Cr > Pb > Cu > As > Cd > Hg. Compared with sediment quality baseline (Table 2) [45,46], Pb and Cu were mostly lower than the TEL (threshold effect level), while other HMs were generally between the TEL and PEL (probable effect level), indicating a low risk of the HMs exerting negative effects on the benthos. Cd and Zn were significantly higher than the soil background values of Guangxi, while other HMs were close to or lower than the soil background values [47]. Therefore, the concentration of Cd and Zn in the sediments of Liujiang River Basin may be elevated by human activities.

The speciations of HMs in sediments are shown in Figure 2 and Table S1. Except for Cd and Pb, HMs were mostly in the residual fraction, indicating that they were derived from crustal materials and less likely involved in the biogeochemical circle of HMs due to the inertia of the minerals containing HMs [48]. More than 80% of As, Cr, and Hg in residual fraction indicated these elements were dominated by natural sources. The proportions of Cu and Zn in the residual fraction were relatively low; both were less than 50%, suggesting a certain amount of anthropogenic input. Pb and Cd were not dominated by a residual fraction but by reducible and carbonate-bound fractions, expressing an intense influence of human activities.

For non-residual fractions, the proportions of exchangeable HMs were extremely low, indicating that the exchangeable fraction was not the main speciation of HMs and should be attributed to the low contents of HMs in water. Zn and Cd were primarily in the carbonate-bound fraction, which is the critical form that preserves exogenous HMs and has higher mobility and bioavailability [48], such that Zn and Cd should be closely related to the emission of exogenous HMs. Pb, Cu, and Cd were relatively high in reducible fractions, which implies that Fe/Mn oxy-hydroxides play a vital role in stabilizing Pb, Cu, and Cd and which is consistent with previous studies that showed Fe/Mn oxy-hydroxides have a strong binding force to Pb, Cu, and Cd and can act as good adsorbents of Pb, Cu, and Cd [49,50,51]. Due to the limited content in sediments, organic matter fails to effectively compete with Fe/Mn oxy-hydroxides and then directly lower the proportions of HMs in the oxidizable fraction. Therefore, the proportions of HMs in the oxidizable fraction were commonly lower than 15%.

### 3.3. The Bioavailability of Heavy Metals in Sediments

**Figure 3:**
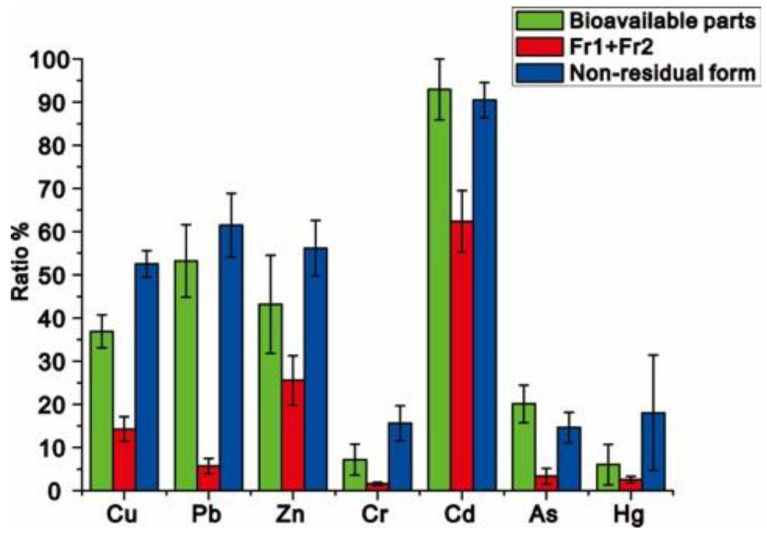
The ratio of heavy metals in bioavailable parts, the exchangeable (Fr1) and carbonate-bound (Fr2) forms, and the non-residual form.

The extraction of the bioavailable heavy metals in the sediments is shown in Figure 3 and Figure S3. The extraction ratio decreased in the order of: Cd > Pb > Zn > Cu > As > Cr > Hg. Among them, Cd was found to be the most bioavailable element, with the extraction ratio of beyond 90%, suggesting a fairly high toxicological effect on aquatic biotas in Liujiang River Basin. Based on our previous studies [52,53], wild fish in these waterways are generally contaminated with Cd, which should unquestionably be blamed on the higher bioavailability of Cd. It is also well-testified that the contamination of Cd in sediments has already exerted its impact on aquatic biotas, so the degradation of Cd bioavailability in sediments should be considered to be the first priority to restore the aquatic environment in Liujiang River Basin. The HMs with moderate toxicological effects were determined to be Pb, Cd, Zn, and As, the extraction ratios of which were 51.97, 45.70, 38.95, and 21.70, respectively. Despite their bioavailability compared to that of Cd, the high bioavailability of these metals should be not overlooked, as their impacts on aquatic biotas are still to be determined. The extraction ratios of Cr and Hg were significantly below 10, which confirmed their lowest toxicological effects on the aquatic environment.

In general, the extraction ratios of bioavailable HMs were significantly higher than that of the exchangeable and carbonate-bound phases but lower than that of the non-residual phase, which illuminated the fact that the bioavailable metals are not confined to the exchangeable and carbonate-bound phases—metals in the reducible and oxidizable fractions should also be incorporated. Because metals in reducible fraction and oxidizable fraction are bioavailable, they can still be digested by aquatic biotas and impair their health to some extent. In other words, the real toxicological effect of HMs in sediments would be greatly underestimated if only concerning metals in the exchangeable and carbonate-bound phases during the assessment of metal pollution in sediments. Therefore, HMs in the reducible and oxidizable fractions should not be overlooked in the assessment of metal pollution. However, ratio gaps between the metals in non-residual forms and their total bioavailability were also found in this study, which suggested that the bioavailability of HMs in the reducible and oxidizable fractions were generally within limits. This means that some inertial parts incorporated into these metal phases are not bioavailable, so metals in non-residual forms that are adopted in the assessment of metal contamination may lead to the overestimation of the real toxicological effect of metals. Thus, researchers should be more rigorous and directly adopt bioavailable HMs instead of their speciations in their pollution judgment.

### 3.4. The Impacts of Heavy Metals Speciation on Their Bioavailability

The correlation between the speciations, total concentration, and bioavailable part of heavy metals is given in Table 3. Except for Hg, the bioavailable HMs were all found to be significantly correlated with their total concentration or specific speciations, which suggested that the total concentration and specific speciations of HMs were substantially responsible for their overall bioavailability. Based on previous studies, the exchangeable phase is the most bioavailable part of HMs, so it should be the most important part to back up their overall bioavailability [23,35,44]; however, this deduction was confirmed to be incorrect, with the correlation coefficients of the exchangeable form found to be lowest on all metals. In fact, compared with other speciations, the ratios of the exchangeable phase are extremely low regardless of the metal, the low ratio of which must directly degrade its contribution to overall bioavailability of HMs in sediments and then lower their correlation coefficients. Based on this analysis, the correlation coefficients of Cu, Pb, and As in the reducible fraction and Zn and Cd in the carbonate-bound fraction were determined to be highest, which corresponded to their highest speciation ratios. At the same time, the correlation coefficients of these metals in other non-residual forms were commonly observed to decrease with the shrinking of the speciation ratios shrinking, which also suggested that the overall bioavailability of metals in sediments should be determined by the ratios of the specific speciations instead of the relative bioavailability of specific speciations themselves. However, the ratio rule may also be out of operation when the form switches to the oxidizable fraction, which Cr well-expressed. The oxidizable fraction is the dominant form of Cr, and it was significantly higher than its other forms. However, the correlation coefficients of this form cannot compare with that of the carbonate-bound and reducible fractions. In fact, metals in the oxidizable fraction not only expressed the metals that combined with organic matter but also contained the metals in the form of metallic sulfides, which were confirmed to be non-bioavailable in previous studies [47,48]. Despite metals that are combined with organic matter being bioavailable, their bioavailability can be mitigated by the excessive existence of metallic sulfides that then degrade the contribution of the oxidizable fraction to the overall bioavailability of metals in sediments, which also results in lower correlation coefficients of oxidizable forms. The excessive existence of metallic sulfides can also impose restrictions on the overall bioavailability of Hg, when, in particular, the oxidizable fraction is its dominant form so that the overall bioavailable ratios of Hg are low and their correlations are non-significant.

Compared with metals in the non-residual form, metals in the residual form are commonly contained in original minerals [17], which are considered to be non-bioavailable. However, the correlations of Cu, Pb, Zn, and As in this form were found to be significant, which seemed to suggest metals in the residual form are not entirely non-bioavailable; however, this may have been related to the weathering of original minerals that is manipulated by microbes that can even release the metals contained in these minerals [49,50]. Given the prevailing anoxic and reducing environment, the metals releasing from original minerals may not directly go back to the water column, instead staying in sediments and turning into non-residual forms. This was supported by the significant correlations between metals in the non-residual and residual forms (Table 4). Therefore, the impacts of metals in the residual form on the overall bioavailability of metal elevation mainly resulted from their transformation instead of their own bioavailability. In addition, it is worth noting that the correlation coefficients of Cu, Pb, Zn, and As were even higher than some of their non-residual forms, but these forms were only in low ratios. Therefore, there is reason to believe that the low ratio of metals in non-residual forms leads to the residual form elevating the overall bioavailability of metals in sediments. Because the weathering rate of minerals depends on their varying composition, more attention should be paid to the elevation degree of potential bioavailability of metals that results from the transformation of their residual form in the future.

### 3.5. The Impacts of Environmental Variations on the Bioavailability of Heavy Metals

Based on our previous analysis, the bioavailability of heavy metals in sediments can be impacted not only by their total contents but their transformation between different speciations. However, these variations of metal bioavailability are primarily triggered by environmental variations [8,23]. Thus, it is essential to analyze the impact of the variations of the physicochemical environment on metal bioavailability. The correlations between environmental parameters (e.g., pH, Eh, DO, EC, TDS, turbidity in water, and organic matter and grain size in sediments) and heavy metals in bioavailable parts were determined in this study (given in Table 5). Among them, only Eh, turbidity, OM, and main grain size (Mz) were found to be correlated with many metals in bioavailable parts, which suggested that these environmental variations are of great importance to regulate metal bioavailability.

Eh is a critical factor that impacts the bioavailability of metals in sediment [23,48]. Here, the accumulation of organic matter and sulfides generally came out in a reducing environment with a low Eh, which resulted in the increasing of metals aggregation in oxidizable form. On the contrary, organic matter and sulfides tend to undergo oxidative decomposition instead of accumulation with an increasing Eh, which primarily influences the stability of metals in the oxidizable form [48]. Therefore, the bioavailability of metals in the oxidizable form was found to be inevitably enhanced with a rise in Eh and then elevated the overall bioavailability of metals, which totally coincided with the significant positive correlations of Eh with Cd, Pb, As, and Zn (Table 5). However, these correlation coefficients did not directly correspond to their ratios in the oxidizable form, which suggested that the impacts that came from pushing up the overall bioavailability of metals may be reserved. In fact, the reducible form was found to be quite stable under oxidation conditions, which was related to the critical speciation of metals that accept metals decomposed from the oxidizable form. Therefore, the transformation between metals in the oxidizable and reducible forms should be treated as the mechanism that leads to the variation of overall metal bioavailability during Eh change. Though the overall bioavailability of metals is elevated with the transformation from metals in the oxidizable form to the reducible form, their elevation degree is closely related to the impacts of their reducible form on their overall bioavailability. The overall bioavailability of Cd, Pb, As, and Zn was determined to be highly supported by their reducible form (Table 3). Their overall bioavailability was significantly elevated with their aggregation in the reducible form, which resulted from the decomposition of their oxidizable form during the rise of Eh. However, the bioavailability of Cu, Cr, and Hg was found to be lower not only in reducible form but also in the oxidizable form, which suggested that their aggregation the reducible form may have trouble exerting its impact on the direct backing up of the overall bioavailability of metals during Eh changes, in particular with the limited supply of metals from the oxidizable form. As a result, the correlations of Cu, Cr, and Hg were determined to be insignificant with Eh. Therefore, despite the fact that metals in the oxidizable form backed up the elevation of metal bioavailability in sediments under fluctuating Eh, the effect of Eh on the bioavailability of metals is more related to their bioavailability in reducible form rather than that in the oxidizable form.

Turbidity is a vital index for suspended particles in water, which are crucial carriers of metal migration and also have significant impacts on metal bioavailability [48,51]. In an aquatic environment, suspended particles are not confined to tiny minerals; instead, they are richer in multisource organic matter [52]. Because organic matter are generally the dominant composition of suspended particles [53], the free metallic ions in overlying water would be substantially suppressed with increases of suspended particles, which results from the absorption of metallic ions in suspended particles. Therefore, the aggregation of exogenous metals in sediments could be heavily suppressed with increases of suspended particles. Because bioavailable metals mainly originate from the exogenous metals, the overall bioavailability of metals is also suppressed by suspended particles in a way that lowers the input of exogenous metals, as confirmed by the significant correlations between Pb, As, Zn, Cu, and turbidity in this study. However, the degree of this suppression is different, and high ratios of metals in the oxidizable form can be partially immune during rising turbidity, while their bioavailability could be heavily suppressed they are in a low ratio, as seen in less significant correlations of Cu and Zn and the higher significant correlations of Pb and As in this study. Therefore, there is reason to believe that metals in the oxidizable form play an important role in regulating the overall bioavailability of metals during turbidity changes. In addition, the correlations between Cr, Hg, Cd, and turbidity were found to be insignificant. For Cr and Hg, despite their oxidizable form being dominant, it still hard to respond to varying turbidity due to the lower bioavailability of their oxidizable form (see Table 3). For Cd, despite its oxidizable form being highly bioavailable, the correlation between Cd and turbidity was still determined to be insignificant in this study, so the large existence of exogenous Cd should be considered to be the critical factor that mitigates turbidity, as confirmed by the extremely high ratio of Cd in the carbonate-bound form.

In addition to Eh and turbidity, other hydrochemical parameters were commonly found to be insignificant for metals, which suggested that the impacts of water chemistry on metal bioavailability should be limited. Despite it being difficult for most hydrochemical variations to exert direct influence on varying metal bioavailability in sediments, they may be treated as effective indicators for the input of exogenous metals, a nice exemplification of which is Cd. The correlations of EC and TDS were found to be significant with total Cd in sediments, which suggested that the dissolved Cd in overlying water could significantly promote the accumulation of Cd in sediments. However, because it is a trace element, the aggregation of Cd in sediments rarely gets support from natural ions in overlying water because the exogenous Cd is largely discharged from sewage. In fact, because the industries in this study area are dominated by electroplating, steel production, and machinery manufacturing [53,54], their sewage is mainly acidic and the concentration of Cd in sewage could be superior to other elements. Therefore, a large emission of the sewage could elevate the input of exogenous metals in sediment and push up the overall bioavailability of Cd while reducing the pH in overlying water at the same time, corresponding to its negative correlation with Cd.

For sediments, the grain size and organic matter are treated as having critical impact on metal bioavailability [41]. The fine grains of sediments contain more organic matter, Fe/Mn oxides, and carbonates with higher specific surface area [6,55], so they generally have more absorption sites for metals ions, which also results in fine grains in sediments commonly having a higher accumulation of metals, particularly in the non-residual phase [53]. Therefore, the more intense aggregation of metals among fine grains pushes up their overall bioavailability, which was confirmed in this study by the negative correlations between bioavailable Cu, Cr, Hg, and mean grain size. Given the more significant increase of metals in the oxidizable form with a rapid elevation of organic matter around fine grains [53], the metals in oxidizable form should be treated as the critical metals form that manipulated the overall bioavailability of metals during grain size changes; however, the existence of metallic sulfide means that the significance of the grain size effect is not directly related to the ratio of metals in the oxidizable form but should be more susceptible to the bioavailability of the oxidizable form (well-expressed in Cu, Cr, and Hg). Their oxidizable forms were all less bioavailable, so their bioavailability could be more easily elevated with the greater aggregation of exogenous metals in fine grains, which was confirmed in this study by the significant correlations with mean grain size. Though the grain size effect is closely related to the bioavailability of metals in the oxidizable form, i the impacts of their ratio on grain size effect should not be overlooked. In fact, the grain size effect is more likely to be aggravated with more metals being preserved in the oxidizable form, as confirmed in this study by the different correlation coefficients of Cu, Hg, and Cr. Higher correlation coefficients were found for Hg and Cu, which corresponded to their high oxidizable form ratio, while the low correlation coefficient of Cr matched its low oxidizable form ratio.

Due to their intense affinity with metals, organic matter us commonly treated as a metal shelter [22,56]. Due to their stable absorption sites, organic matter can directly aggravate the aggregation of exogenous metals [22] and then elevate the overall bioavailability of metals in sediments, which is consistent with the significant correlations between bioavailable Cu, Cr, Hg, and organic matter found in this study. Given that metals combined with organic matter are the critical components of metals in the oxidizable form, the overall variation of metal bioavailability could be connected to such a form. However, similar to the grain size effect, the existence of metallic sulfide cuts off the direct connection between the ratio of metals in the oxidizable form and their overall bioavailability during organic matter changes, so the bioavailability of metals in the oxidizable form should be treated as a critical factor on organic matter effects. Cu, Cr, and Hg were all found to be less bioavailable in their oxidizable form, so the effect of the organic matter should only be more significant if the bioavailability of metals in the oxidizable form is within limits, which may be related to the sensitivity between the aggregation of exogenous metals and their bioavailability. Though the effect of organic matter could be dominated by the bioavailability of metals in oxidizable form, the impacts of their ratios should not be overlooked, as can be seen in the higher correlation coefficients of Hg and Cu and the lower correlation coefficients of Cr in this study.

Due to the widespread weathering of carbonate rocks in the karst area, there are excessive carbonate ions in karstic rivers, so the exogenous HMs can predominantly preserve as a carbonate-bound fraction [17,30], which, however, makes them susceptible to variations of pH and ion concentration in a water column because this form of HMs is weakly bonded [17,22]. Therefore, fluctuations of pH, EC, and TDS raised misgivings about the surge of HM bioavailability in previous studies [17,22]. Though the combination of HMs in the carbonate-bound phase is weak, the excessive carbonate ions and alkalescent water in karstic rivers could be treated as the shields of this weakly bonded form of HMs [57] that decrease the impact of environmental variations. In fact, the correlations between pH, EC, TDS, and HM bioavailability were commonly found to be insignificant in this study, which demonstrated that HMs in the carbonate-bound phase are relatively stable and not sensitive to environmental variations. On the contrary, given that HMs in reducible form and oxidizable form were found to be heavily affected by Eh, turbidity, OM, and Mz, the fluctuation of HM bioavailability under changes of these factors is probably primarily connected to the regulation of HMs in the reducible and oxidizable forms, i.e., though combinations of HMs in the reducible and oxidizable forms are relatively intense and less bioavailable, they are susceptible to environmental fluctuation. Therefore, there is reason to believe that the regulation of HM bioavailability in karstic rivers is closely related to HMs in the reducible and oxidizable forms, rather than the carbonate-bound form.

## 4. Conclusions

HMs in sediments were all found to be within the PEL, but Cd and Zn were significantly higher than the soil baseline. Most HMs were in the residual fraction, while their exchangeable fraction was in an extremely low ratio. HMs in bioavailable parts were significantly higher than that in the exchangeable and carbonate-bound phases but lower than those in the non-residual phase, which demonstrated that HM bioavailability is not confined to the exchangeable and carbonate-bound phases. The correlation coefficients commonly decreased with decreasing speciation ratios, which suggested that the overall bioavailability of metals should be determined by speciation ratios instead of the speciations themselves. Noteworthily, most HMs in the residual form significantly correlated with their overall bioavailability, which demonstrated the potential bioavailability of the residual form. The non-correlations between pH, EC, TDS, and HM bioavailability suggested that HMs in the carbonate-bound phase are stable and unsusceptible to environmental variations. However, the significant correlations between Eh, turbidity, OM, Mz, and HM bioavailability suggested that HMs in the reducible and oxidizable forms are susceptible to environmental fluctuations. Therefore, the variation of HM bioavailability in karstic rivers is largely regulated by their reducible and oxidizable forms instead of their carbonate-bound form.

## Figures and Tables

**Table 1:** The properties in sediments and their overlying water. EC: electrical conductivity; DO: dissolved oxygen; Eh: redox potential; TDS: total dissolved solids; OM: organic matter; Mz: main grain size.

	DO (mg/L)	EC (μs/cm)	pH	Eh (mV)	TDS (ppm)	Turbidity
Overlying water chemistry						
Min–Max	6.22–8.43	141.5–252.2	6.79–8.48	94.89–161.1	71.1–127.2	8.13–28.1
Mean	7.2	168.1	7.81	114.03	84.9	13.61
	OM (%)	Mz (μm)	Sand (%)	Silt (%)	Clay (%)	
Sediment properties						
Min–Max	0.37–1.37	10.24–27.63	0.05–7.24	62.74–79.84	15.74–27.63	
Mean	0.72	21.23	3.79	74.05	22.16	

**Table 2:** Heavy metal concentrations in sediments. BSG: background values of soil in Guangxi, China; TEL: threshold effect level; probable effect level.

Location	Cd	Pb	Cr	Cu	Zn	As	Hg
mg/kg							
BSG	0.267	24	82.1	27.8	75.6	20.5	0.152
Liujiang River Basin	0.44–6.36	17.74–43.31	27.64–91	19.98–35.86	68.47–196.95	8.29–69.76	0.09–1.32
	1.27	30.10	53.53	25.20	124.93	23.24	0.19
TEL	0.6 ^a^	35 ^a^	42 ^a^	36 ^a^	123 ^a^	7.2 ^b^	0.17 ^a^
PEL	3.5 ^a^	91 ^a^	160 ^a^	197 ^a^	315 ^a^	42 ^b^	0.49 ^a^

^a^. The freshwater sediment quality of Canada; ^b^. The sediment quality criteria of Hong Kong.

**Table 3:** The correlation between the bioavailable part, speciations, and total concentration of heavy metals.

	BCu	BPb	BZn	BCr	BCd	BAs	BHg
Fr1	0.244	0.182	0.466 *	0.309	0.696 **	0.424 *	0.061
Fr2	0.511 *	0.887 **	0.970 **	0.538 **	0.998 **	0.793 **	0.295
Fr3	0.720 **	0.970 **	0.958 **	0.732 **	0.982 **	0.947 **	0.202
Fr4	0.599 **	0.882 **	0.817 **	0.455 *	0.872 **	0.612 **	0.232
Fr5	0.668 **	0.426 *	0.681 **	0.069	0.369	0.842 **	−0.125
Total	0.839 **	0.957 **	0.959 **	0.135	0.998 **	0.842 **	−0.08

**. Correlation is significant at the 0.01 level (2-tailed). *. Correlation is significant at the 0.05 level (2-tailed). Note: Fr1–Fr5 means metals in exchangeable fraction, carbonate-bound fraction, reducible fraction, oxidizable fraction, and residual fraction, respectively; Bmetal is equal to metal in bioavailable part.

**Table 4:** The correlation of heavy metals between metals in non-residual and residual forms.

	Cr	Cu	Zn	Cd	Pb	As	Hg
	F5						
F1	0.640 **	0.593 **	0.448 *	0.201	0.234	0.163	0.781 **
F2	0.361	0.185	0.672 **	0.372	0.225	0.622 **	0.063
F3	0.366	0.647 **	0.606 **	0.383	0.467 *	0.892 **	0.068
F4	0.616 **	0.407	0.741 **	0.423 *	0.623 **	0.905 **	−0.032

**. Correlation is significant at the 0.01 level (2-tailed). *. Correlation is significant at the 0.05 level (2-tailed). Note: Fr1–Fr5 means metals in exchangeable fraction, carbonate-bound fraction, reducible fraction, oxidizable fraction, and residual fraction, respectively.

**Table 5:** The correlation between heavy metals in bioavailable part and environmental variations.

	Do	Ec	pH	Eh	TDS	Turbidity	OM	Mz
BCu	0.257	0.213	0.054	0.286	0.215	−0.466 *	0.768 **	−0.559 **
BPb	0.366	0.318	−0.021	0.602 **	0.317	−0.631 **	0.255	−0.244
BZn	0.286	0.3	0.063	0.511 *	0.298	−0.522 *	0.175	−0.274
BCr	0.291	0.325	0.089	0.053	0.324	−0.306	0.455 *	−0.449 *
BCd	0.114	0.737 **	−0.500 *	0.738 **	0.734 **	−0.308	0.035	−0.033
BAs	0.209	−0.057	0.209	0.526 **	−0.058	−0.539 **	−0.082	0.027
BHg	0.136	0.302	−0.321	0.01	0.303	−0.149	0.797 **	−0.526 **

**. Correlation is significant at the 0.01 level (2-tailed). *. Correlation is significant at the 0.05 level (2-tailed). Bmetal is equal to metal in bioavailable part.

## Data Availability

The original contributions presented in the study are included in the article, further inquiries can be directed to the corresponding author.

## Supplementary Materials

The following are available online at https://www.mdpi.com/article/10.3390/ijerph18083986/s1, Figure S1: The spatial properties in the overlying water., Figure S2: The spatial properties in sediments., Figure S3: The bioavailable ratios of heavy metals in sediments., Table S1: Heavy metal levels (mg/kg dry weight) in sediments with different fractions.
