# Supplementary material for: The Variation of Heavy Metals Bioavailability in Sediments of Liujiang River Basin, SW China Associated to Their Speciations and Environmental Fluctuations, a Field Study in Typical Karstic River
Source: Int J Environ Res Public Health. 2021 Apr 10;18(8):3986. doi: 10.3390/ijerph18083986 (PMC8069464; doi:10.3390/ijerph18083986)
Supplement: Supplementary file 1 [file ijerph-18-03986-s001.pdf]

Article

The Variation of Heavy Metals Bioavailability in Sediments of Liujiang River Basin, SW China Associated to Their Speciations and Environmental Fluctuations, A Field Study in Typical Karstic River

Yupei Hao [1], Xiongyi Miao [1,2]*, Hongwei Liu [3] and Dan Miao [4]

[1] Key Laboratory of Karst Dynamics, MNR&GZAR, Institute of Karst Geology, CAGS, Guilin, 541004, China
[2] The Second Engineering Investigation Institute of Guizhou Bureau of Geology and Mineral Exploration and Development, Zunyi 563000, China
[3] Anhui Province Key Laboratory of Polar Environment and Global Change, Department of Environmental Science and Engineering, University of Science and Technology of China, Hefei 230026, China
[4] Department of Chemistry and Environmental Engineering, Wuhan Bioengineering Institute, Wuhan 430415, China
* Correspondence: miaoxy88@126.com (X.M.)

Figure S1. The spatial properties in the overlying water.

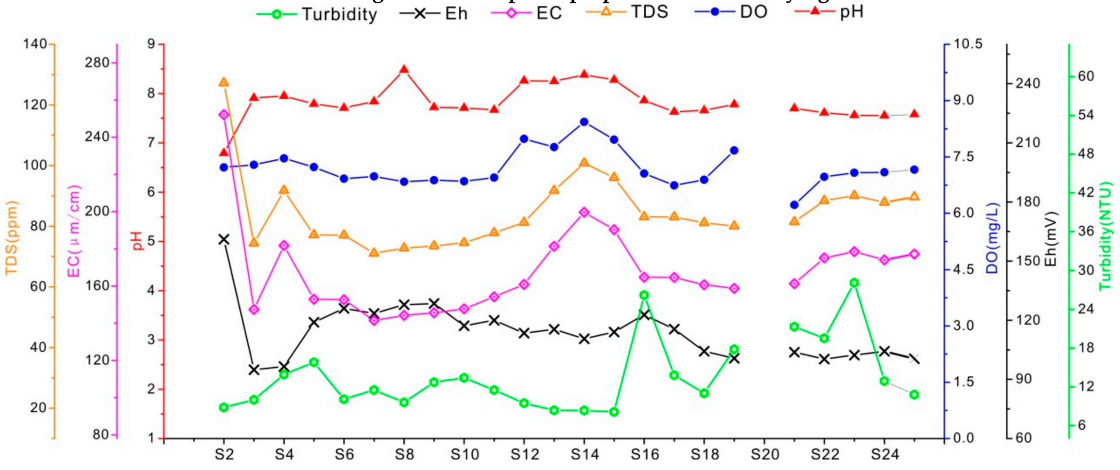

Figure S2. The spatial properties in sediments.

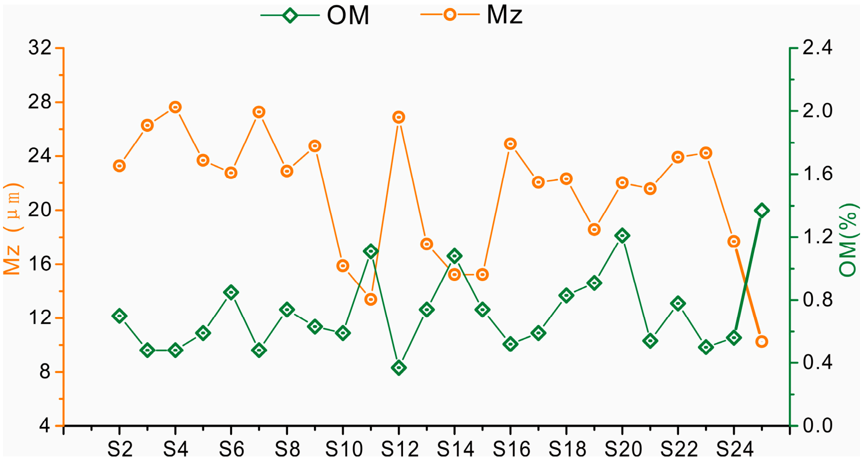

Table S1. Heavy metal levels (mg/kg dry weight) in sediments with different fractions.

Metal	Exchangeable Fraction (mg/kg)		Carbonate-bound Fraction (mg/kg)		Reducible Fraction (mg/kg)		Oxidizable Fraction (mg/kg)		Residual Fraction (mg/kg)	
	Mean±SD	Ratio(%)	Mean±SD	Ratio(%)	Mean±SD	Ratio(%)	Mean±SD	Ratio(%)	Mean±SD	Ratio(%)
**Cr**	0.28±0.09	0.53	0.53±0.09	1.04	2.12±0.68	4.12	5.14±1.16	9.98	45.64±13.53	84.32
**Cu**	0.64±0.29	2.53	2.91±0.78	11.63	6.5±1.51	25.68	3.21±0.64	12.79	11.91±1.86	47.36
**Zn**	0.57±0.27	0.47	33.91±16.32	25.64	19.63±9.4	14.85	19.16±4.31	15.66	53.05±11.84	43.38
**Cd**	0.01±0.01	0.95	0.95±1.13	61.43	0.29±0.29	20.57	0.08±0.02	7.51	0.09±0.02	9.54
**Pb**	0.05±0.04	0.15	1.72±0.79	5.45	16.15±6.14	51.53	1.3±0.31	4.3	11.18±1.34	38.57
**As**	0.01±0	0.08	0.72±0.46	3.25	1.52±0.8	6.73	1.22±1.64	4.3	19.22±10.24	85.64
**Hg**	0.0012±0.0005	0.34	0.0024±0.0008	1.73	0.0068±0.0018	5.14	0.02±0.05	12.47	0.15±0.22	79.85

Figure S3. The bioavailable ratios of heavy metals in sediments.

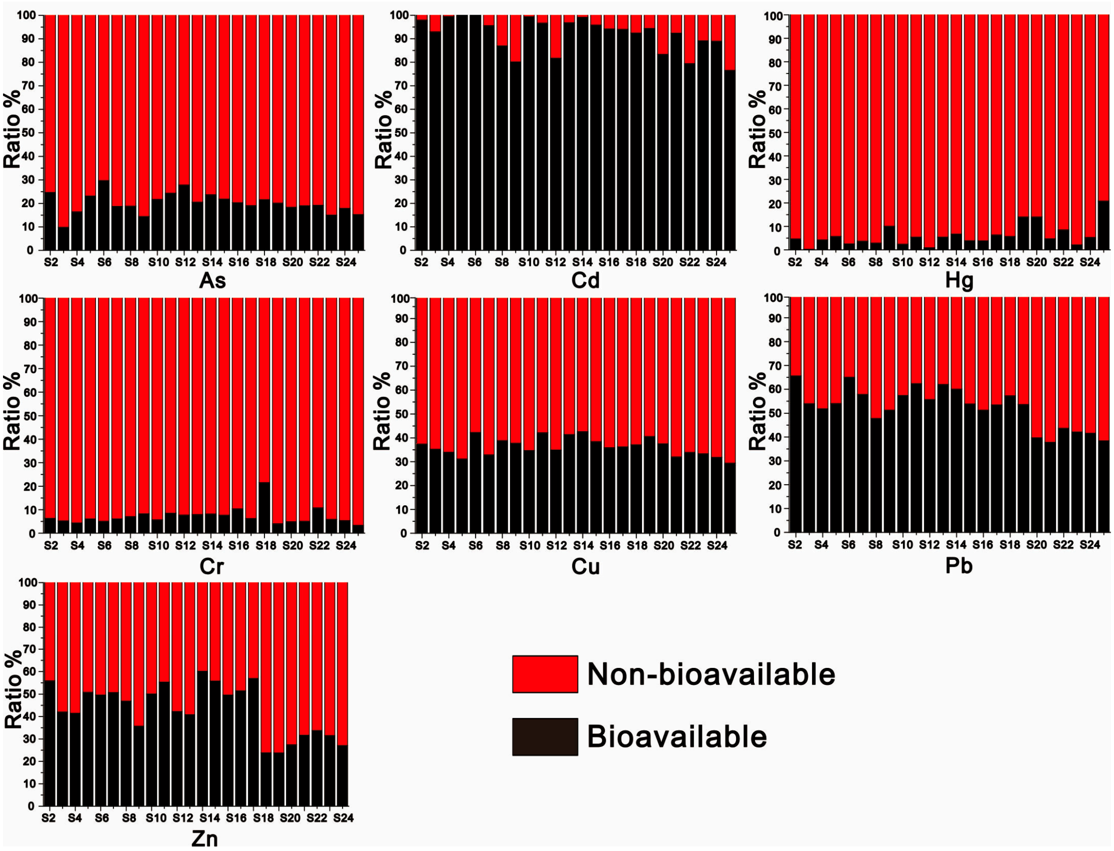

.
